# Asbestos and erionite prime and activate the NLRP3 inflammasome that stimulates autocrine cytokine release in human mesothelial cells

**DOI:** 10.1186/1743-8977-10-39

**Published:** 2013-08-13

**Authors:** Jedd M Hillegass, Jill M Miller, Maximilian B MacPherson, Catherine M Westbom, Mutlay Sayan, Joyce K Thompson, Sherrill L Macura, Timothy N Perkins, Stacie L Beuschel, Vlada Alexeeva, Harvey I Pass, Chad Steele, Brooke T Mossman, Arti Shukla

**Affiliations:** 1 Department of Pathology, University of Vermont College of Medicine, Burlington, VT, USA; 2 Langone Medical Center, NYU School of Medicine, New York, NY, USA; 3 Department of Medicine, University of Alabama at Birmingham School of Medicine, Birmingham, AL

**Keywords:** Asbestos, Mesothelioma, Mesothelium, Inflammasomes, NLRP3

## Abstract

**Background:**

Pleural fibrosis and malignant mesotheliomas (MM) occur after exposures to pathogenic fibers, yet the mechanisms initiating these diseases are unclear.

**Results:**

We document priming and activation of the NLRP3 inflammasome in human mesothelial cells by asbestos and erionite that is causally related to release of IL-1β, IL-6, IL-8, and Vascular Endothelial Growth Factor (VEGF). Transcription and release of these proteins are inhibited *in vitro* using Anakinra, an IL-1 receptor antagonist that reduces these cytokines in a human peritoneal MM mouse xenograft model.

**Conclusions:**

These novel data show that asbestos-induced priming and activation of the NLRP3 inflammasome triggers an autocrine feedback loop modulated via the IL-1 receptor in mesothelial cell type targeted in pleural infection, fibrosis, and carcinogenesis.

## Background

Human mesothelial cells (HMC) possessing phenotypic and functional features of both epithelial cells and fibroblasts are unique cell types lining the pleural (lung), peritoneal and pericardial cavities. They are target cells of asbestos-associated malignant mesothelioma (MM), unique and devastating tumor types with a poor prognosis. For example, the average life span of patients with MM is approximately 12 months after diagnosis, despite a number of therapeutic strategies (reviewed in [1-3]). The incidence of MM is increasing worldwide, especially in third world countries where use of asbestos is largely unregulated; thus MM remains a global health problem. In addition, abnormal function of mesothelial cells is intrinsic to the development of pleural effusions after infection or pleural injury and pleural fibrosis, an asbestos-associated disease with no effective treatment options [4].

Mechanisms of pulmonary injury that contribute to the development of pulmonary fibrosis (asbestosis) by asbestos fibers have been studied for decades in clinical and experimental studies (reviewed in [5,6]). The hallmarks of asbestos inhalation in humans and rodents include early and sustained inflammation causally attributed to initial accumulation of alveolar macrophages that attempt to engulf asbestos fibers, participate in mucociliary clearance of fibers from the lung, and transport fibers to lymph nodes or the lung interstitium. A number of studies have focused on chemotactic factors and cytokine/chemokine release by alveolar macrophages that are essential to neutrophil influx, macrophage-epithelial or macrophage-fibroblast signaling, and the development of epithelial cell hyperplasia and fibrosis. We initially reported mechanisms of activation of the Nod-like receptor-family protein 3 (NLRP3, NALP3) inflammasome and IL-1β production by asbestos fibers in human macrophage-like cells (THP-1 line) and monocytes *in vitro*[7]. These data support a model in which long asbestos fibers, known to be more inflammatory, carcinogenic and fibrogenic than shorter fibers in rodents (reviewed in [5,6,8]), elicited reactive oxygen species through iron-driven redox reactions on the fiber surface as well as by prolonged NADPH oxidase stimulation during frustrated phagocytosis of fibers. Use of NLRP3 knock-out mice showed that the inflammasome was linked functionally to pulmonary inflammation and elevations in IL-1β and KC, a potent neutrophil chemoattractant, in bronchoalveolar lavage fluids. Moreover, NLRP3 knockout mice were more resistant to asbestos-induced lung injury. Subsequently, inflammasome activation by a myriad of endogenous and exogenous stress-associated danger signals (DAMPs), including infectious and toxic agents, has been intensely studied in myeloid cells and very little in non-myeloid cells. This work has generally focused on modulation of the immune system, properties and mechanisms of agents affecting inflammasome activation, and signaling pathways/receptors involved (reviewed in [9,10]).

Although macrophages play critical roles in clearance as well as inflammation and cell signaling in response to pathogenic fibers in the airways and interstitium of the lung, little is known about whether or not they are required for translocation of asbestos fibers to the pleura or are essential for pleural inflammation and genesis of pleural diseases (reviewed in [8]). For example, accumulation of fibers in localized acellular areas (pleural plaques) is observed on the pleural surface as opposed to fiber-laden alveolar macrophages and asbestos bodies (iron-coated fibers linked to accumulation and death of macrophages) in the lung [1,5,6,8].

Here we tested the hypothesis that activation of the NLRP3 inflammasome occurs in human mesothelial cells after pleural injury and is linked causally to release of critical cytokines associated with the development of inflammation and pleural diseases. First, we show that *in vitro* exposures to the naturally occurring, mineralogically distinct fibers crocidolite asbestos and erionite (a fiber type linked to epidemic proportions of MM in areas of Turkey) [11], cause increases in transcription of NLRP3 mRNA and release of mature IL-1β that are inhibited using NLRP3 siRNA. These changes were accompanied by increases in caspase-1 activity. Elevations in steady-state mRNA levels of NLRP3, IL-1β, IL-6 and IL-8, and release of IL-1β, IL-6, IL-8 and VEGF by HMC were causally linked to an autocrine pathway that was inhibited after addition of the IL-1 receptor antagonist (IL-1ra), Anakinra. In addition, we demonstrate that fiber-exposed HMC cells release the alarmin, HMGB1, via a NLRP3-dependent pathway that is abrogated by blocking the IL-1 receptor (IL-1r). Lastly, we used a well-characterized human xenograft model of peritoneal MM [12] to show early (1 and 4 wks) production of critical cytokines in peritoneal lavage fluid (PLF) by human MM prior to tumor establishment. Cytokines (IL-8, VEGF, IL-6) in PLF were inhibited most markedly at 1 wk after intraperitoneal (IP) injection of Anakinra in the absence of changes in numbers of macrophages, neutrophils or lymphocytes. Our studies highlight the functional importance of inflammasome-mediated cytokine production via an autocrine pathway in HMC that is perpetuated by durable pathogenic fibers in the pleura. Moreover, data reveal that mesothelial cells are pluripotent cells responding to fiber-induced NLRP3 activation by producing inflammasome-associated pro-inflammatory and angiogenic cytokines via an autocrine feedback loop. We did not observe a significant reduction in spheroid/tumor volume after 4-wks of once daily Anakinra treatment. This may be due to the fact that Anakinra has very short half-life in mice. Future experiments might require a constant infusion of Anakinra for it to become effective. Taken together, our *in vitro* and *in vivo* results suggest that selective targeting of the NLRP3 inflammasome or IL-1r may be critical in the prevention and therapy of asbestos-induced pleural diseases.

## Results

### Asbestos causes NLRP3 priming and activation in human mesothelial cells

**Figure 1:**
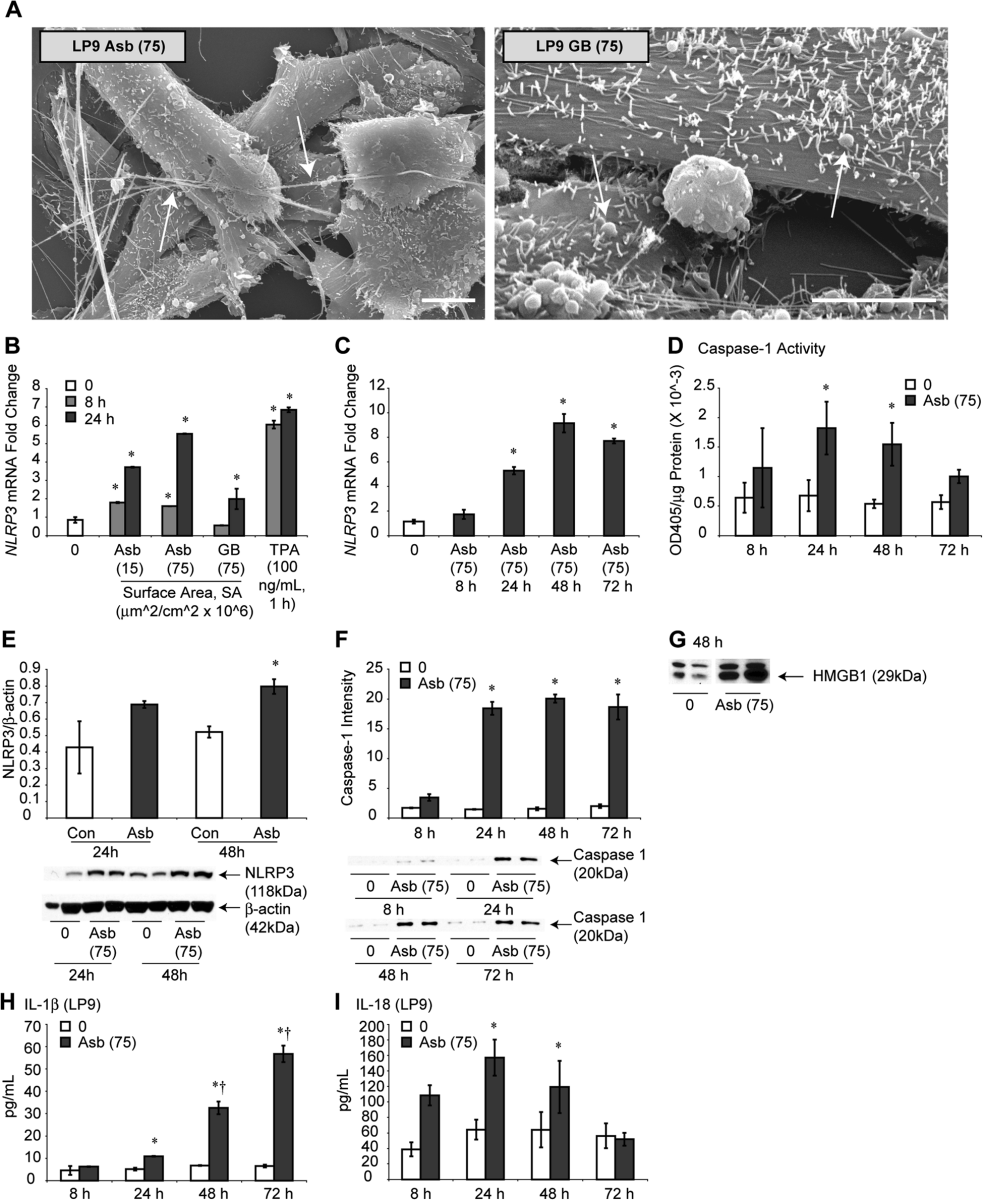
**Asbestos primes and activates *NLRP3 *in HMC. (A)** Crocidolite asbestos fibers (arrows in left panel) and GB (arrows in right panel) interact with differentiated LP9 mesothelial cells that are characterized by long microvilli both *in vivo* and *in vitro.* Bars = 10 μm. **(B)** Crocidolite asbestos (Asb) and the soluble tumor promoter, TPA cause increased trends in NLRP3 mRNA levels, as demonstrated by qRT-PCR. In contrast, non-pathogenic GB at identical SA concentrations have no effects (N = 2 samples/group/time point; N = 3 for control, 8 + 24 h (0) group). **(C)** A time course study shows the protracted nature of *NLRP3* transcription by asbestos (75) (N = 2 samples/group/time point; N = 4 for control (0)). **(D)** Caspase-1 activity is significantly increased by asbestos (75) at 24 and 48 h (N = 4 samples/group/time point; combined data from two experiments). A randomized block ANOVA was used to determine significance for this experiment. * = significantly different p ≤ 0.05) from untreated control group (0). **(E)** Asbestos-induced NLRP3 protein levels in HMC. **(F)** Western blot analysis of secreted p20 subunit of caspase-1 (an indicator of caspase-1 activation) in medium in response to asbestos exposure in HMC (N = 2 samples/group/time point). **(G)** HMGB1 release in medium from HMC cells in response to asbestos exposure. **(H)** IL-1β released into the medium of HMC in response to asbestos exposure as measured by ELISA, and IL-18 **(I)** released over time as measured by ELISA (N = 2 samples/group/time point). * = significantly different (p ≤ 0.05) from 0 control at same time point; † = significantly different (p ≤ 0.05) from 8 h asbestos group.

Crocidolite asbestos (Na_2_O · Fe_2_O_3_ · FeO · 8SiO_2_ · H_2_O) is considered to be the most pathogenic of the several asbestos types in the induction of MM [1,2]. To determine if HMC expressed the NLRP3 inflammasome and whether its transcription occurred selectively in response to pathogenic fibers, we first exposed the LP9-TERT-1 (LP9) HMC line to crocidolite asbestos in a dose–response experiment over a 24 h period, i.e., the time necessary for precipitation of fibers on cells. The soluble tumor promoter, 12-O-tetraoctodecanol phorbol-3 acetate (TPA) (added for 1 h) was included as a positive control, and amorphous glass beads (GB) as a non-pathogenic particle control. In comparison to untreated control cells, both asbestos and TPA caused increased expression of NLRP3 mRNA in contrast to GB (Figure 1B). Increased transcription of NLRP3 by asbestos was protracted (Figure 1C), an observation of direct relevance to mechanisms of action of durable, pathogenic fibers in the lung and pleura over time. NLRP3 protein was also increased by asbestos exposure (Figure 1E). We then measured caspase-1 activity, an inflammasome-activation phenomenon linked to processing of mature IL-1β in HMC in the presence and absence of asbestos fibers (Figure 1D). These studies revealed that caspase-1 activity was significantly elevated (p ≤ 0.05) by asbestos as measured by activity assay and Western blot analysis to show p20 release in supernatant (medium) (Figure 1F). A consequence of inflammasome activation is release of mature IL-1β, produced as an inactive cytosolic precursor that is regulated and released by caspase-1. IL-1β is a critical protein facilitating inflammation, production of other pro-inflammatory cytokines, and mesothelial cell transformation (reviewed in [9,10,13]). In Figure 1H, we show dramatic release of IL-1β over time by asbestos in LP9 mesothelial cells. In addition, we show increased levels of HMGB1, and IL-18 release in the medium from asbestos-exposed cells (Figure 1G,I). As the ELISA kits used for IL-1β and IL-18 detection detect predominantly mature forms, we assume that asbestos-induced NLRP3 activation is processing these two cytokines and mature form is being detected in medium. These studies in concert show that protracted NLRP3 transcription by asbestos is accompanied by caspase-1 activation. Moreover, transcription is selectively induced by a fibrous (asbestos) or soluble tumor promoter (TPA) and not by inert particles (GB). Both asbestos and GB interacted with and were taken up by HMC *in vitro* (Figure 1A). To confirm that increased NLRP3 mRNA levels after asbestos exposure were due to transcriptional regulation, we pre-treated LP9 cells with Actinomycin D (Act D) that abolished these elevations (Additional file 1: Figure S1A). Next we validated our findings on inflammasome priming and activation by asbestos in human primary peritoneal mesothelial cells (HM3). Additional file 1: Figure S1B shows that like immortalized human mesothelial cells (LP9), asbestos fibers also prime and activate NLRP3 in HM3 cells. Primary peritoneal HM3 mesothelial cells also demonstrate significant secretion of IL-1β in response to asbestos exposure (Additional file 1: Figure S1C).We also assessed the effect of asbestos on other inflammasomes NLRP1 and Absent in Melanoma 2 (AIM2) and on PYCARD/ASC and found no significant effect (data not shown).

### Asbestos causes other cytokine release from mesothelial cells

To confirm and determine whether IL-1β and other cytokines were released in both LP9 and NYU474 primary pleural HMC, we performed Bio-Plex cytokine assays on medium from these cells (24 h after asbestos exposure). Tumor Necrosis Factor-α (TNFα) was added to some groups to determine whether or not it was a necessary factor for asbestos-induced cytokine release. The rationale for these studies is that TNFα is mitogenic to mesothelial cells [14], causes MM cell transformation [13], and may have pleiomorphic effects on inflammation and/or cell defense as it is linked to both upregulation of the mitochondrial antioxidant enzyme, manganese-containing superoxide dismutase (SOD2) [15], as well as increased chemokine production by lung macrophages and neutrophils after pathogenic particle exposures [16].

**Figure 2:**
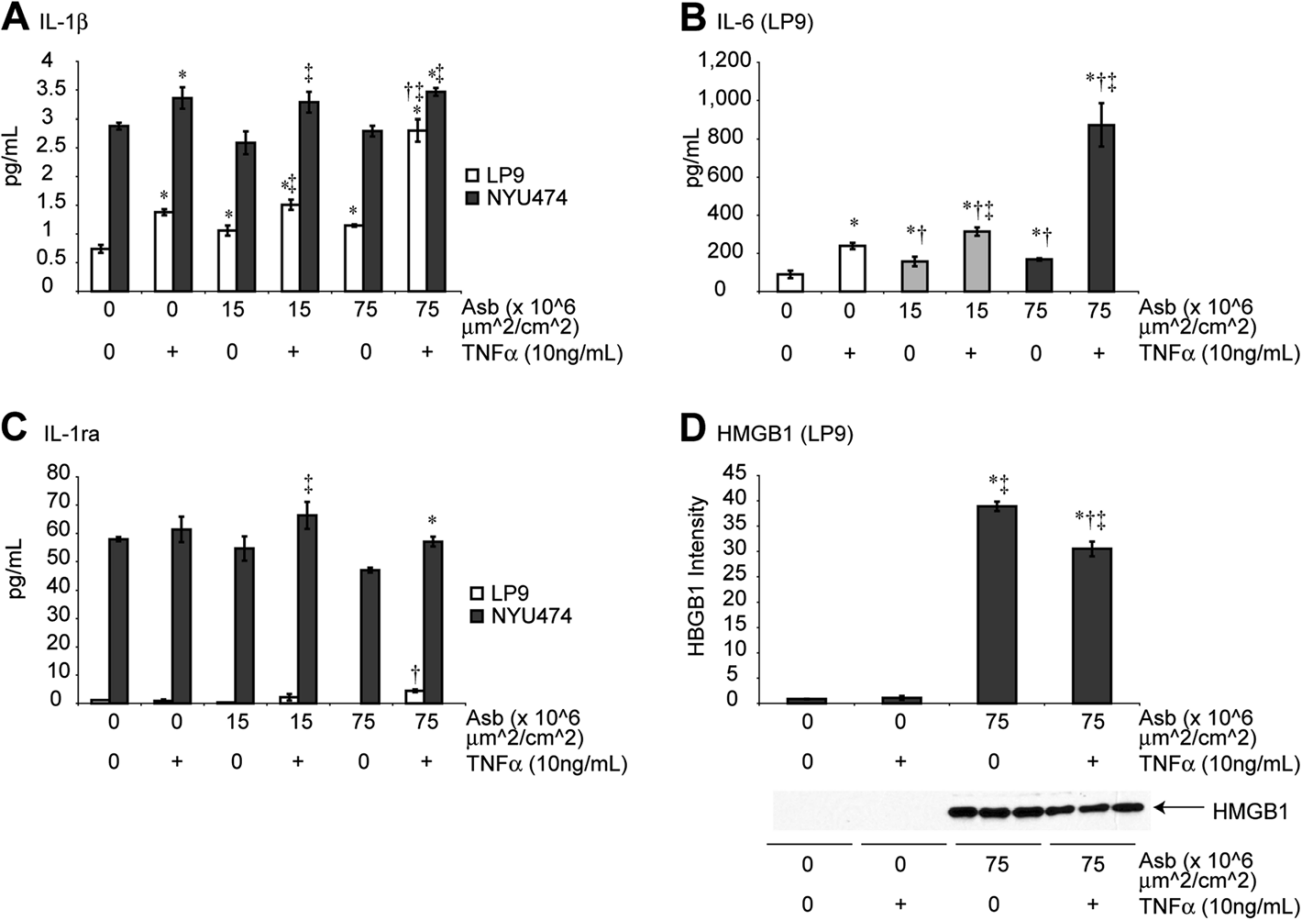
**Asbestos and TNFα causes release of cytokines from HMC.** The LP9 cell line and isolated pleural mesothelial (NYU474) cells in culture were exposed to asbestos in the presence (+) or absence (0) of TNFα. **(A)** Release of IL-1β in the presence and absence of TNFα. **(B)** Levels of IL-6 in medium. IL-6 levels in NYU474 cells were elevated and above the scale of detection for LP9 cells. **(C)** Levels of IL-1ra in medium. All experiments in **A**-**C** were performed using a Bio-Plex protein array system for 27 human cytokines on media supernatants collected after 24 h of exposure to agents (N = 3 samples/group). Levels of IL-8 in groups of NYU474 and LP9 cells were higher than the standard curve of detection. **(D)** TNF priming did not increase asbestos-induced HMGB1 release from LP9 cells at 48 h (N = 3 samples/group/timepoint). * = significantly different (p ≤ 0.05) from untreated control group (0); † = significantly different (p ≤ 0.05) from TNFα-treated group; ‡ = significantly different (p ≤ 0.05) from asbestos alone group at the same concentration.

Similar trends of IL-1β release from LP9 and NYU474 HMC were observed in that addition of either TNFα (10 ng/mL) or asbestos alone at non-toxic or lytic (15 vs. 75 × 10^6^ μm^2^/cm^2^) surface area (SA) concentrations [17], significantly (p ≤ 0.05) increased IL-1β levels in medium (Figure 2A). These experiments also revealed that TNFα and high dose of asbestos acted synergistically on IL-1β release from LP9 cells (The differences in levels of IL-1β detected in two different experiments, Figures 1H and 2A, could be attributed to different techniques (ELISA kit and Bio-Plex cytokine assay) used for assessment). The important point is asbestos exposure causes significant increases in IL-1β secretion from mesothelial cells. In LP9 mesothelial cells, significantly (p ≤ 0.05) increased IL-6 levels were observed after addition of asbestos or TNFα alone, and dramatic synergy was observed with use of both agents, especially at the higher concentration of asbestos (75) (Figure 2B). Levels of IL-1ra produced in medium by mesothelial cells were increased significantly only in cells exposed to both asbestos and TNFα (Figure 2C). In contrast, TNFα had no effect on HMGB1 release from LP9 cells (Figure 2D) and TNFα and asbestos together did not produce a synergistic effect on HMGB1 release (Figure 2D).

**Figure 3:**
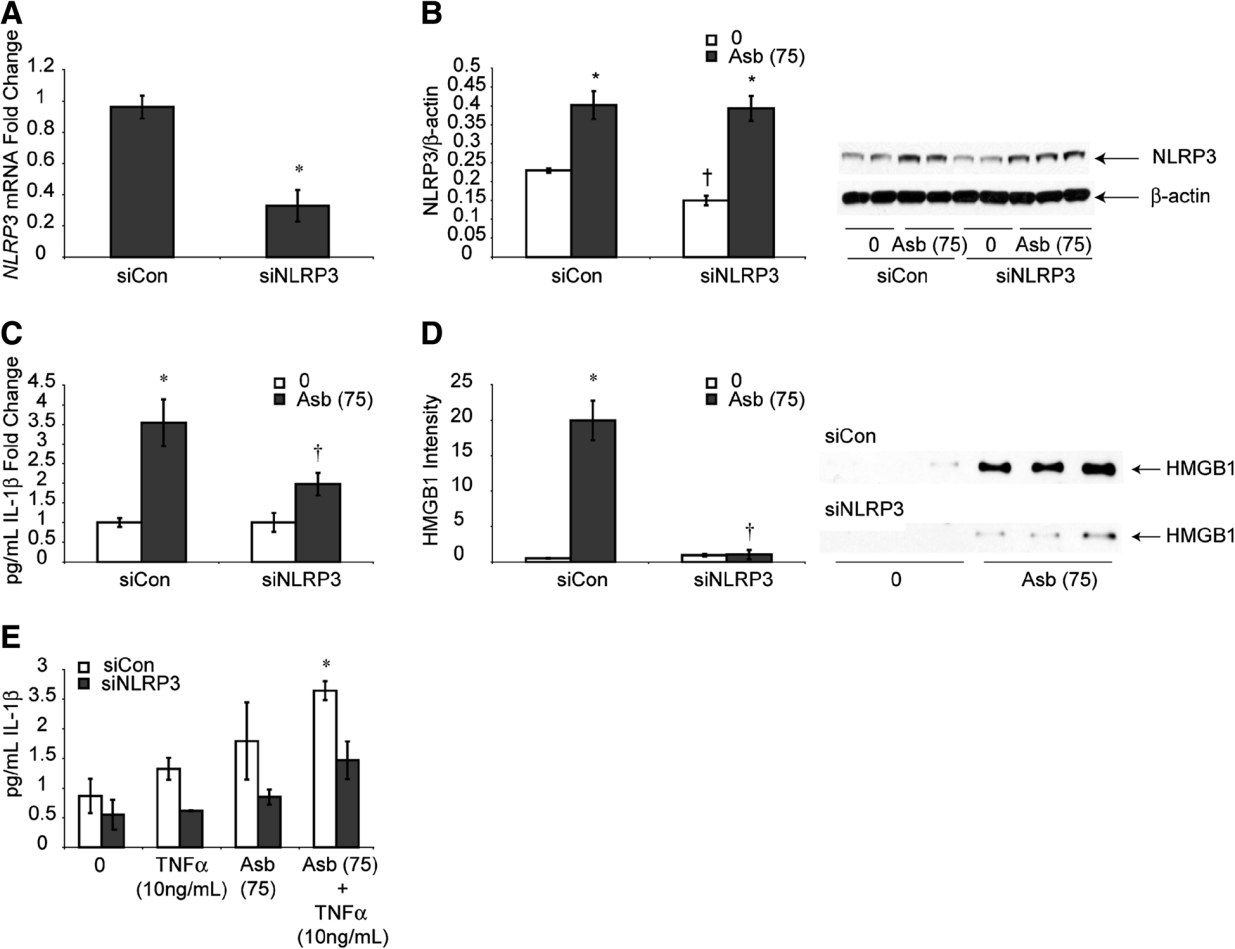
**NLRP3 inhibition attenuates IL-1β and HMGB1 release from HMC. (A)** Inhibition of NLRP3 using siNLRP3 in LP9 cells significantly inhibits siNLRP3 mRNA levels (* = p ≤ 0.05 as compared to siCon group) and **(B)** Inhibition of NLRP3 using siNLRP3 in LP9 cells significantly attenuated NLRP3 protein levels at 78 h. **(C)** Production of mature IL-1β, (* = p ≤ 0.05 as compared to untreated siCon cells; † = p ≤ 0.05 as compared to siCon asbestos-exposed group). **(D)** Crocidolite asbestos increases HMGB1 release that is blocked in siNLRP3-transfected cells at 24 h (N = 3 samples/group). * = significantly different (p ≤ 0.05) when compared to siCon group; † = significantly different (p ≤ 0.05) when compared to siCon asbestos-exposed group. Priming effect of TNFα on IL-1β was blocked by siNLRP3 at 48 h **(E)** (N = 2, 4 for asbestos alone groups, * = significantly different (p ≤ 0.05) from siCon untreated (0) group).

To determine whether NLRP3 activation was causally related to release of IL-1β, confirming an inflammasome-activated pathway in HMC, downregulation of NLRP3 was verified in siNLRP3- transfected LP9 cells in contrast to cells transfected with scrambled control siRNA (siCon) (Figure 3A). Protein levels of NLRP3 showed significant but lesser in magnitude effect may be because of slow turnover rate of NLRP3 protein in LP9 cells (Figure 3B). This approach also showed that asbestos-induced increases in IL-1β and HMGB1 were decreased significantly (p ≤ 0.05) in siNLRP3-transfectants (Figure 3C,D). Inhibition of NLRP3 also attenuated priming effect of TNFα and asbestos on IL-1β (Figure 3E). However, knock-down of NLRP3 did not affect asbestos-induced cell death (data not shown).

### NLRP3 activation by erionite

**Figure 4:**
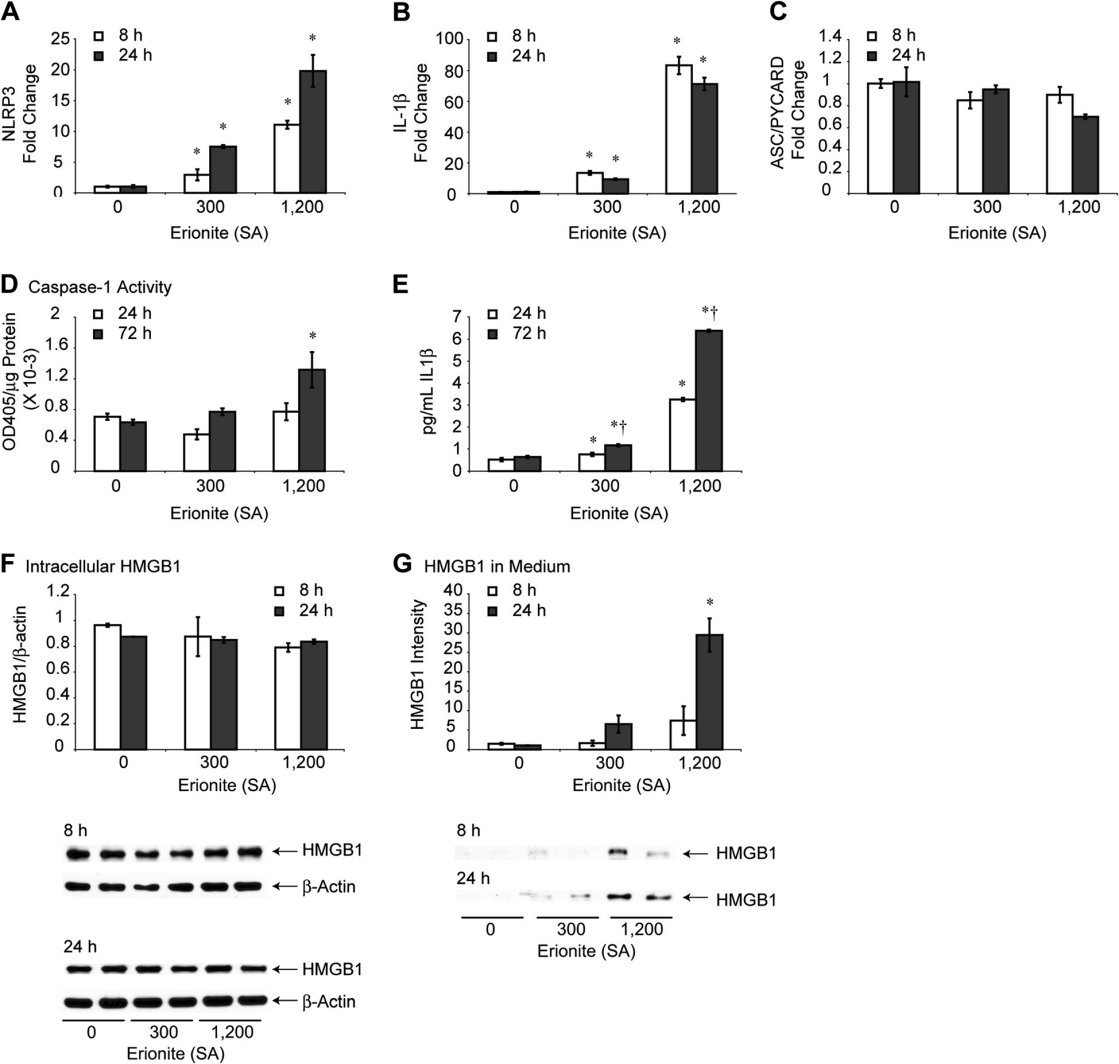
**NLRP3 priming and activation by erionite in HMC.** Human mesothelial cells (LP9) exposed to erionite *in vitro* show dose-related increases in steady-state mRNA levels of NLRP3, **(A)** and IL-1β, **(B)** but not ASC/PYCARD **(C)**. Increases in caspase-1 activity **(D)** and IL-1β release **(E)** also are observed by erionite at high concentrations (SA = 1,200 μm^2^/cm^2^ dish) (N = 3 samples/group/time point). * = significantly different (p ≤ 0.05) from untreated control group (0) at same time point; † = significantly different (p ≤ 0.05) from respective erionite-exposed group at 24 h. Lack of intracellular accumulation of HMGB1 in response to erionite **(F)** supports rapid dose-related increases in release by erionite as measured by Western blot analyses on cell medium **(G)** (N = 2 samples/group/time point). * = significantly different (p ≤ 0.05) as compared to untreated control group (0) at the same time point.

Here we tested the hypothesis that erionite fibers primed and activated the inflammasome. First, we performed dose–response toxicity studies in LP9 cells to find that erionite fibers over a range of SA concentrations as high as 300 × 10^6^ μm^2^/cm^2^ were non-toxic to cells over a 72 h period. This lack of cell death at high concentrations of fibers contrasted markedly with the toxicity of crocidolite asbestos at 75 × 10^6^ μm^2^/cm^2^ (Additional file 2: Figure S2C). These data support previous reports showing that erionite fibers are more mesotheliomagenic than asbestos because progenitor cells of MM are not killed by erionite fibers. A subsequent toxicity study with erionite revealed that concentrations as high as 1,200 × 10^6^ μm^2^/cm^2^ resulted in significant (p ≤ 0.05) decreases in cell numbers at 24 h that did not increase over a time period of 72 h (Additional file 2: Figure S2D). As shown in Figure 4A-C, erionite at both non-toxic (300) and toxic (1,200) amounts caused dose-related increases in steady-state mRNA levels of *NLRP3* and *IL-1β,* but not the adapter-interacting protein (*ASC/PYCARD*), a component of the multi-protein inflammasome platform. Caspase-1 activity was elevated significantly by erionite (1,200) at 72 h (Figure 4D), whereas dose-related increases in release of IL-1β were observed by erionite at 300 and 1,200 SA concentrations (Figure 4E). The resistance of mesothelial cells to erionite-induced toxicity and erionite’s ability to cause activation of the NLRP3 inflammasome as well as production of IL-1β and other cytokines at non-toxic concentrations may be of mechanistic significance to promulgation of erionite-transformed cells and its increased potency in MM carcinogenesis.

HMGB1 is a putative biomarker of inflammation in a number of diseases including endotoxemia, cystic fibrosis, and some tumors including MM [18-20]. In the presence of IL-1β or TNFα, this nuclear protein is released into the cell cytoplasm or secreted. Once extracellular, HMGB1 binds to a number of cytokine proteins that confer increased pro-inflammatory activity [21]. To determine whether erionite caused increased intracellular levels of HMGB1 or its immediate release via an NLRP3 inflammasome-dependent process, LP9 cells were exposed to erionite (SA = 300, 1,200). These studies revealed that although increases in intracellular levels of HMGB1 were not observed at 8 or 24 h by erionite (Figure 4F), toxic concentrations of erionite (1,200) caused significantly elevated levels of HMGB1 in medium (Figure 4G). This finding suggests that although erionite does not increase the expression of HMGB1, it does alter its release from cells.

### Anakinra alters cytokine transcription

**Figure 5:**
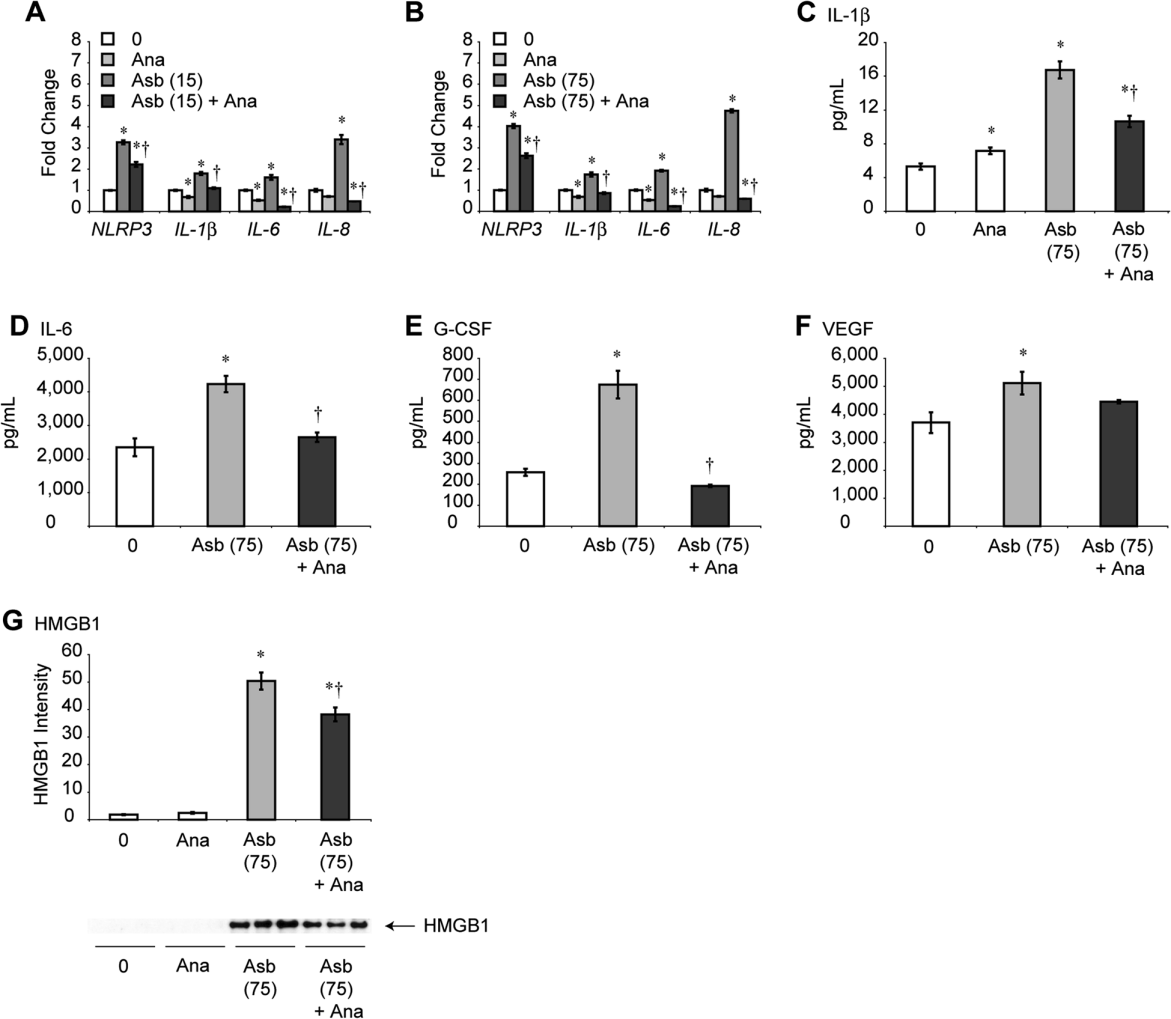
**Anakinra inhibits asbestos-induced NLRP3 and interleukin mRNA levels and secretion of cytokines in medium.** Use of the IL-1ra, Ana (100 ng/mL), decreases asbestos-induced **(A**, **B)** mRNA levels of *NLRP3* and *IL-1β*, *IL-6*, and *IL-8* at 24 h, as quantitated by qRT-PCR (N = 3 samples/group/time point). * = significantly different (p ≤ 0.05) when compared to untreated control group (0); † = significantly different (p ≤ 0.05) when compared to asbestos group alone. Asbestos-induced secretion of IL-1β **(C)** IL-6 **(D)** G-CSF **(E)** and VEGF **(F)** were measured in LP9 cells at 24 h using a human IL-1β ELISA kit **(C)** or a human cytokine Bio-Plex assay **(D**-**F)** in the presence and absence of Ana (Ana = 100 ng/mL). Levels of HMGB1 **(G)** were determined by Western blot analysis (N = 3 samples/group). * = significantly different (p ≤ 0.05) when compared to untreated cells (0); † = significantly different (p ≤ 0.05) when compared to groups exposed to asbestos alone.

We next hypothesized that transcription and release of IL-1β (and possibly other cytokines linked to interaction of IL-1β or IL-1α with the IL-1 receptor) would be altered after pre-addition of Anakinra (Ana) to HMCs. After performing a series of dose–response studies with Anakinra *in vitro* and *in vivo* at concentrations used by others [22,23], we performed studies using Anakinra at 100 ng/mL, a non-toxic dose causing significant decreases in elevated mRNA levels of *NLRP3*, *IL-1β*, *IL-6* and *IL-8* observed at both low and high concentrations of asbestos (Figure 5A,B). Next we evaluated whether Anakinra pre-treatment significantly inhibited release of asbestos-induced IL-1β, IL-6, granulocyte-colony stimulating factor (G-CSF) [24], VEGF and HMGB1. In most cases, asbestos-exposed cells showed levels of cytokines in medium that were lower (p ≤ 0.05) after pre-addition of Anakinra (Figure 5C-G). These results suggest that an autocrine pathway exists in mesothelial cells whereby blocking of the IL-1 receptor renders less transcriptional upregulation of asbestos-induced NLRP3 and NLRP3-regulated cytokines as well as their production and release by cells.

### Anakinra alters cytokines in PLF of SCID mice

**Figure 6:**
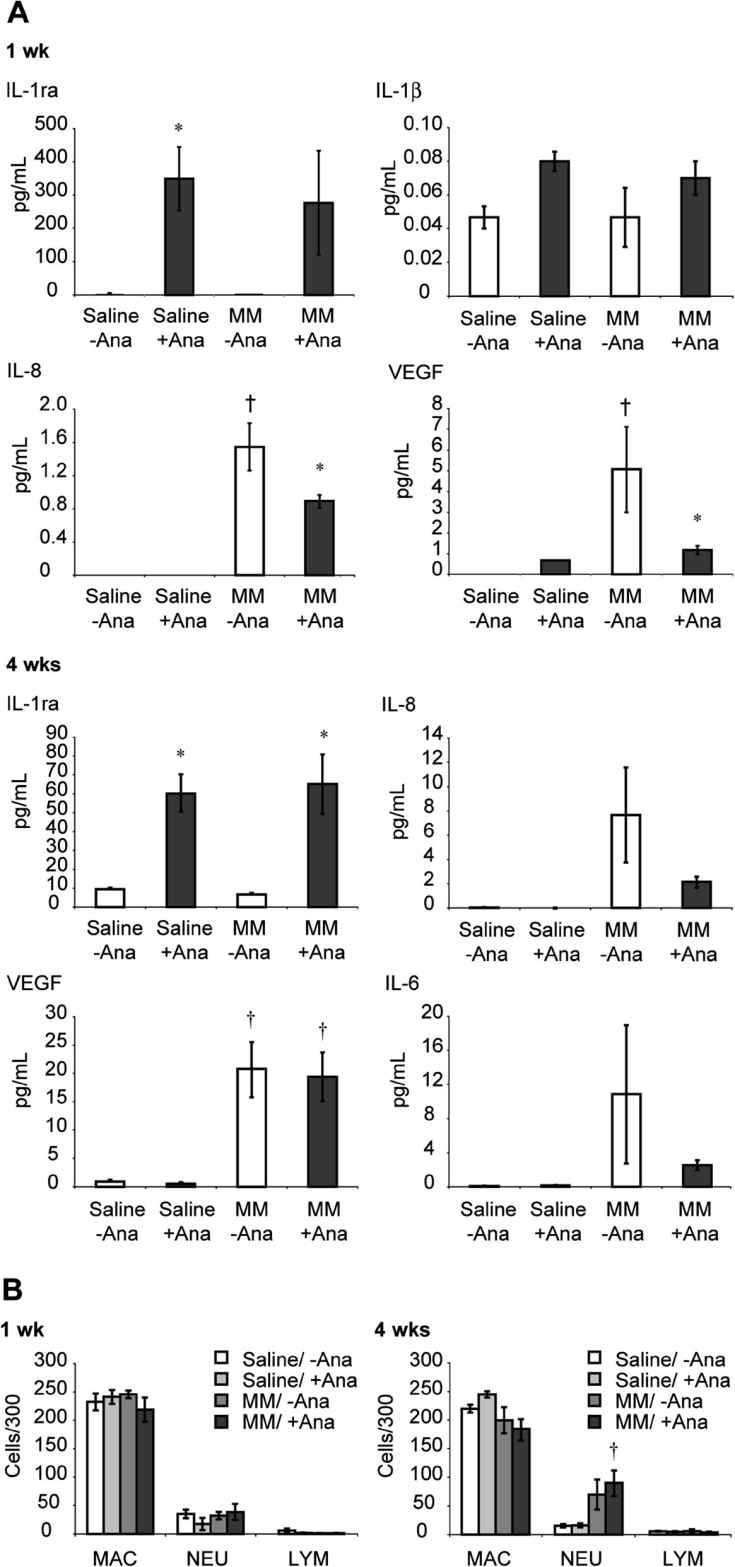
**Anakinra inhibits MM-induced cytokines in PLF of SCID mice. (A)** at 1 wk, N = 3 samples/group; at 4 wks, N = 5–6 samples/group). * = significantly different (p ≤ 0.05) when compared to respective groups receiving no Anakinra; † = significantly different (p ≤ 0.05) when compared to respective saline (no tumor cell) groups. Left panels at 1 and 4 wks verify activity of Anakinra, an IL-1ra, in respective treatment groups. Use of a Bio-Plex panel for mouse cytokines on the same PLF samples failed to reveal any significant changes [12]. **(B)** Lack of changes in macrophages, neutrophils and lymphocyte numbers and proportions at 1 and 4 wks suggests the absence of involvement of cells of the immune system in cytokine release and inflammation. † = significantly different (p ≤ 0.05) when compared to respective saline (no Ana) groups.

As a next relevant step in demonstrating the ramifications of this feedback loop in a model of peritoneal MM, we determined whether injection of Ana blocked pro-inflammatory cytokine and VEGF levels in PLF from severe combined immunodeficient (SCID) mice after injection of human H2373 MM cells. These mice are ideal for our studies as they have normal macrophages and neutrophils, but are deficient in lymphocyte function which permits human MM establishment. In this model, early increases in IL-6, IL-8 and VEGF in lavage fluids are produced by both epithelioid and sarcomatoid MM after their injection into mice that precede tumor development [12]. Ana (100 mg/kg) was administered to mice receiving saline alone (no tumor cells) and immediately following MM cell injection in saline. PLF was collected from these and control groups not receiving Ana at 1 and 4 wks for detection of human cytokines and total and differential cell counts. As presented in Figure 6A, Ana significantly (p ≤ 0.05) reduced levels of IL-8 and VEGF in PLF at 1 wk. At 4 wks, mice injected with Ana showed trends in inhibition of IL-6 and IL-8 levels; however, differences did not reach statistical significance because of large variability in the MM group not receiving Ana. Likewise, no significant decreases in VEGF or IL-8 levels by Ana were observed at this later time point, presumably because of overwhelmingly (approximately 4 and 7-fold, respectively) increased production of these factors by human MM from 1 to 4 wks (no significant differences in IL-1β levels at any time point in any group were observed, 4 wks data not shown). Total and differential cell counts in PLF revealed no significant differences in numbers of macrophages, neutrophils or lymphocytes between Ana-treated and non-Ana-treated mice at either time point, although increased neutrophils were observed in all MM-injected mice at 4 wks as reported previously [12] (Figure 6B). The lack of early inflammatory responses in all groups suggests that MM cells may not require cells of the immune system for cytokine release and inflammation.

## Discussion

Most research on the role of inflammation in asbestos-associated diseases has focused on macrophages, as this is the first cell type accumulating in the lung at sites of initial deposition of inhaled asbestos fibers. In addition, the vast majority of published studies with few exceptions [25] exploring the role of inflammasomes in the development of a number of diseases have been performed in macrophages or monocytes. Erionite, a non-asbestos fiber type of the zeolite mineral group, is morphologically similar to crocidolite asbestos (Additional file 2: Figure S2A, B), but is more potent in the causation of MM in rodents [26] and is of contemporary concern because of the high prevalence of MM observed in areas of Turkey where erionite fibers exist in soils and homes [11,27]. The uniqueness of work here is the demonstration that the pathogenic fibers, asbestos and erionite, but not innocuous particles, such as glass beads, cause both priming and activation of the NLRP3 inflammasome in human mesothelial cells, responses that trigger transcription and production of cytokines critical to the initiation of pleural injury and infection, MM and pleural fibrosis. These responses were validated in primary human pleural and peritoneal mesothelial cells. Most importantly, we link NLRP3 activation to the release of several pro-inflammatory cytokines and VEGF by fiber-stimulated human mesothelial cells *in vitro*. As these factors are identical to those produced from human MM during the initial stages of inflammation in a SCID mouse MM xenograft model, we believe that IL-1β may be responsible for MM tumor progression also in absence of asbestos fibers. Our studies support a model where an autocrine loop in human mesothelial cells is perpetrated by fiber-induced NLRP3 priming and activation and increased transcription of *IL-1β*, *IL-6* and *IL-8* (Additional file 2: Figure S2E). Under these circumstances, HMGB1 may bind to these cytokines, thus increasing its pro-inflammatory properties [21] and be exuded into serum after injury to the pleura. In support of our findings Yang et al. [20] have previously shown the release of HMGB1 from asbestos-exposed mesothelial cells, however, we are first to link the asbestos-induced HMGB1 release and NLRP3. Findings of Yang et al. link HMGB1 release as a critical first step in the pathogenesis of asbestos-associated diseases. Both NF-κB and mitogen-activated protein kinase signaling have been implicated as mechanisms regulating the transcription of pro-IL-1β [28], and asbestos activates both of these pathways in mesothelial cells [29-31]. Transcription and production of IL-1β may be key to induction of VEGF gene expression at both transcriptional and post-transcriptional levels [32] as well as IL-6 and IL-8 expression [33]. IL-6 also induces VEGF production through a JAK2/STAT3 pathway [34] and can modulate production of IL-1β in a JAK2-dependent manner [35]. Thus, other pathways of synergy and interaction between these factors may exist in HMC in addition to increased expression and release of these cytokines via inflammasome and caspase-1 activation [36].

The main purpose of our study here is to demonstrate that stimulation of the IL-1 receptor may be essential for cytokine induction (via an autocrine loop) by asbestos- exposed mesothelial cells in initiation of MM (*in vitro* studies) as well as in MM tumor progression (*in vivo* studies). Our *in vitro* studies show that mesothelial cells secrete IL-1β in response to asbestos/erionite which can then affect the same population of mesothelial cells in an autocrine manner, thus transforming them to become mesothelioma cells. Our *in vivo* model on the other hand demonstrates that MMs generated from mesothelial cells after asbestos exposures can further secrete IL-1β and/or be affected by IL-1β secreted by host cells, thus affecting their growth/progression (in the absence of asbestos). Therefore, blocking the IL-1 receptor *in vivo* could be helpful in attenuation of MM growth and progression.

An exciting observation in both *in vitro* studies and our human MM xenograft model of inflammation was that levels of interleukins and VEGF were blocked using the IL-1r antagonist, Anakinra, currently used for clinical treatment of various inflammatory diseases, including Muckle-Wells syndrome [37], gout [38], and arthritis [39]. The lack of significant effects of Anakinra on IL-6, IL-8 and VEGF levels at 4-weeks (Figure 6) could be attributed to two reasons: 1) overwhelming increases in levels of VEGF (4 fold) and IL-8 (7 fold) at 4-weeks; and 2) the short half-life of Anakinra in mice (~4 hours). Future experiments will include repeated dosing or constant infusion of Anakinra to produce significant effects. Our results suggest an autocrine feedback loop resulting in cytokine release that can be blocked at the IL-1 receptor in mesothelial cells. In addition to abrogating the inflammatory mechanisms of mature IL-1β that may also orchestrate the expression, production, and effects of IL-6, IL-8 and VEGF, this approach may be valuable in preventing the ramifications of robust production and release of IL-1 from necrotic and neighboring mesothelial cells after infection [40] or exposure to asbestos fibers. IL-1α is a key danger signal that triggers both CXCL1 secretion and recruitment of neutrophils via interaction with the IL-1 receptor in murine mesothelial cells [40]. The fact that Anakinra is non-toxic after repeated administration to mice or humans [23] suggests its use as a novel approach in prevention and treatment of inflammation, a common feature of pleural infection and disease.

## Conclusions

In summary, data here question the popular paradigm that initiation of pleural diseases by pathogenic fibers requires cells of the immune system. Moreover, they reveal that unlike macrophages and monocytes, and like MSTO-211H human mesothelioma cells [25], human mesothelial cells do not require exogenous TNFα or lipopolysaccharide to initiate NLRP3-mediated cytokine release. Conversely, exposure to pathogenic fibers both primes and activates the NLRP3 inflammasome in these cell types.

## Methods

### Cell cultures

LP9/TERT-1 (LP9), an hTERT-immortalized cell line that functionally resembles normal human peritoneal mesothelial cells [41] and human primary peritoneal mesothelial cells (HM3) were obtained from Dr. James Rheinwald (Brigham and Women’s Hospital, Harvard University, Boston, MA). Human pleural mesothelial cells (NYU474) were isolated surgically from cancer-free patients by Dr. Harvey I. Pass (New York University, New York, NY), as was the H2373 sarcomatoid MM cell line [42]. The H2373 MM line gives rise to biphasic MM after injection into SCID mice as verified by a board-certified pathologist (Dr. Kelly Butnor at UVM) [12]. LP9 and H2373 cells were cultured as described previously [17]. NYU474 cells were grown to near confluence in DMEM containing 10% FBS and supplemented with penicillin (50 units/mL) and streptomycin (100 μg/mL). HM3 cells were grown in 50:50 M199:MCDB106 medium (Invitrogen, Carlsbad, CA) supplemented with 15% FBS, 10 ng/mL EGF, 0.4 μg/mL hydrocortisone, 50 units/mL penicillin and 100 μg/mL streptomycin. All cells were incubated at 37°C in 5% CO_2_ and grown to 80-90% confluence before addition of agents.

### Sources of crocidolite asbestos, erionite and glass beads

The physical and chemical characterization of the NIEHS reference sample of crocidolite asbestos has been reported previously [43]. Erionite fibers from Pine Valley, NV, were provided and characterized using similar methods by Dr. Ian Steele (University of Chicago, Chicago, IL). The SA of asbestos and erionite fibers and GB (Polysciences Inc., Warrington, PA) was measured using nitrogen gas sorption analysis. Asbestos, erionite fibers and glass beads were exposed to UV light overnight to inactivate any pathogens growing on them before adding to cell culture.

### Scanning electron microscopy (SEM)

Cells grown on Thermonox coverslips (Nalge Nunc International, Naperville, IL) were fixed for imaging as previously described [17], and critical point-dried using liquid CO_2_ as the transition fluid in a Samdri PVT-3B critical point dryer (Tousimis Research Corporation, Rockville, MD). Cells on coverslips and fibers on carbon tape were then mounted on aluminum specimen stubs and dried before being sputter-coated with gold and palladium in a Polaron sputter coater (Model 5100; Quorum Technologies, Guelph, ON, Canada) and examined using a JSM 6060 scanning electron microscope (JEOL USA, Inc., Peabody, MA).

### Viability studies

Following sterilization under ultraviolet light overnight to destroy lipopolysaccharide or microbial contaminants, minerals were suspended in 1 X Hanks’ Balanced Salt Solution (HBSS) at 1 mg/mL, sonicated for 15 min in a water bath sonicator, and triturated 5 X through a 22-gauge needle. This suspension was added to cells in medium to achieve the desired SA-based concentrations. After 24 h intervals, cells were collected by trypsinization, and counted using a hemocytometer [17].

### Quantitative Real-Time PCR (qRT-PCR)

Total RNA (1 μg) was reverse-transcribed with random primers using the Promega AMV Reverse Transcriptase kit (Promega, Madison, WI) according to the recommendations of the manufacturer as described previously [17]. Transcription was evaluated using the ∆∆Ct method. Duplicate or triplicate assays were performed with RNA samples isolated from at least two independent experiments. The values obtained from cDNAs and hypoxanthine phosphoribosyl transferase (*hprt*) controls provided relative gene expression levels for the gene locus investigated. The primers and probes used were purchased from Applied Biosystems (Foster City, CA).

### Western blot analysis for NLRP3, activated Caspase-1 p20 and HMGB1 in whole cell lysates and supernatants (medium) of asbestos-exposed mesothelial cells

Medium was collected, cells were lysed as previously described [44], and protein content in cell lysates was determined using the RC DC protein assay (Bio-Rad, Hercules, CA). Medium (500 μL) was concentrated using Amcion^®^ ultracentrifugal filters with a 10 K membrane (Millipore, Billerica, MA) by spinning at 14,000 X g for 30 min. Columns were then reversed into new collection tubes and spun for 2 min at 1,000 X g. Sample buffer was added to the concentrated supernatant, and samples were boiled for 15 min. Western blots were performed as previously described [44] on both cell lysates (NLRP3, HMGB1) and concentrated supernatants (HMGB1, Capase1-p20). Rabbit polyclonal antibodies for HMGB1 (Abcam, Cambridge, MA), NLRP3 (Novus Biologicals, Littleton, CO) and Caspase-1-p20 (Cell Signaling, Danvers, MA) were used at 1:500 dilutions.

### Caspase-1 activity assay

Caspase-1 activity was measured using the Caspase-1 Colorimetric Assay (R&D Systems, Minneapolis, MN), according to the manufacturer’s directions. Caspase-1 activity was determined as OD405 (after subtraction of a blank control) relative to total protein. Protein concentrations were determined by the Bio-Rad protein assay [45] using the remaining lysate.

### ELISA assay for IL-1β and IL-18

The Quantikine Human IL-1β/IL-1f2 Immunoassay (R&D Systems, Minneapolis, MN, measures predominantly mature IL-1β) was used on concentrated cell medium, prepared as described earlier, and the assay performed according to the manufacturer’s instructions. A total of 500 μL of cell supernatant was concentrated. Two hundred μL samples with assay diluents were loaded into 96 well plates pre-coated with IL-1β antibody. For IL-1β release studies in erionite-exposed cells, asbestos exposed HM3 cells and TNFα priming of siCon and siNLRP3 LP9 cells, an ELISA MAX™ Human IL-1β (Biolegend, San Diego, CA, measures both pro and mature form) was used. Wells were pre-coated with an IL-1β capture antibody overnight at 4°C. Fifty μL of concentrated samples or standards then were prepared in assay diluent and allowed to attach to plates overnight at 4°C. IL-18 release was measured using the Human IL-18 ELISA kit (MBL International, Woburn, MA, measures predominantly active IL-18, 0.7% pro IL-18). Medium was collected (500 uL), concentrated and processed according to the ELISA protocol provided. Values were expressed as pg/mL of IL-1β or IL-18 from the original supernatant (non-concentrated).

### Transfection of cells with NLRP3 siRNA

On-Target plus non-targeting siRNA #1 (scrambled control), and On-Target plus SMART pool human NLRP3 siRNA (100 nM; Dharmacon, Lafayette, CO) were transfected into LP9 cells at near confluence using Lipofectamine 2000 (Invitrogen, Carlsbad, CA) following the manufacturer’s protocol. The efficiency of NLRP3 knockdown was determined by qRT-PCR.

### SCID mouse xenograft model, IP injection of human MM, and retrieval of PLF

H2373 cells (5 × 10^6^ cells in 50 μL 0.9% NaCl, pH 7.4) were injected into the peritoneal cavity of 6 wk-old male Fox Chase SCID mice (*n* = 3 mice/group/time point) as described previously [12]. Mice were euthanized using sodium pentobarbital before the peritoneal cavity of each mouse was instilled with 5 mL of cold sterile PBS using an 18-gauge needle. The abdomen was gently massaged, and PLF was aspirated back into the syringe and placed on ice. PLF was then centrifuged at 1,000 rpm for 5 min at 4°C, and the supernatant removed and stored at -80°C for Bio-Plex cytokine analysis. Cytospins were prepared from cell pellets to determine total and differential cell counts [12].

### Bio-Plex assays on cell medium in vitro and in PLF

To quantify cytokine and chemokine levels in cell supernatants and PLF, a multiplex suspension protein array was performed using a Human Cytokine 27-plex panel (Bio-Rad) as described previously [12]. Concentrations of each cytokine and chemokine were determined using Bio-Plex Manager Version 3.0 software. Data were expressed as pg cytokine/mL medium.

### In vitro and in vivo studies using the IL-1-ra, Ana

For *in vitro* experiments, cells were pre-treated with 100 ng/mL of IL-1ra Ana (Insight Genomics, Falls Church, VA) for 1 h before administration of asbestos [22]. For *in vivo* experiments, SCID mice either injected with 500 μL sterile 0.9% NaCl (pH 7.4) or human MM cells (H2373) in saline were then injected with 100 mg/kg Ana (Kineret^®^) (Amgen, Thousand Oaks, CA) in 500 μL sterile 0.9% NaCl (pH 7.4) [23] or 500 μL sterile 0.9% NaCl (pH 7.4) alone. Mice were injected IP daily (with saline or Ana) for 1 wk observations or 3 X IP weekly for 4 wks before euthanization and collection of PLF for Bio-Plex cytokine analyses and cytospin preparations as described above.

### Statistical methods

Data were evaluated either by analysis of variance (ANOVA) using the Student Neuman-Keul’s procedure for adjustment of pair-wise comparisons between groups or a Student’s *t*-test. Differences in gene expression values determined by qRT-PCR were evaluated using a Student’s *t*-test. Differences with p values ≤ 0.05 were considered statistically significant. All experiments were repeated in duplicate or triplicate. Figures represent individual experiments, unless otherwise noted, with Mean ± SEM presented.

## Abbreviations

MM: Malignant Mesotheliomas; VEGF: Vascular endothelial growth factor; HMC: Human mesothelial cells; NLRP3 NALP3: Nod-like receptor-family protein 3; THP-1 line: Human macrophage-like cells; DAMPs: Endogenous and exogenous stress-associated danger signals; IL-1ra: IL-1 receptor antagonist; IL-1r: IL-1 receptor; PLF: Peritoneal lavage fluid; IP: Intraperitoneal; TPA: 12-O-tetraoctodecanol phorbol-3 acetate; GB: Glass beads; Act D: Actinomycin D; HM3: Human primary peritoneal mesothelial cells; LP9: Immortalized human mesothelial cells; TNFα: Tumor Necrosis Factor-α; SOD2: Superoxide dismutase; SA: Surface area; siCon: Scrambled control siRNA; Ana: Anakinra; MSU: Monosodium urate crystals; G-CSF: Granulocyte-colony stimulating factor; SCID: Severe combined immunodeficient; SEM: Scanning electron microscopy; HBSS: Hanks’ Balanced Salt Solution; qRT-PCR: Quantitative Real-Time PCR; hprt: Hypoxanthine phosphoribosyl transferase; ANOVA: Analysis of variance; Asb: Crocidolite asbestos; Act D: Actinomycin D.

## Competing interests

The authors declare no competing interests.

## Authors’ contributions

AS conceived, designed and supervised the experiments, analyzed the data and wrote the manuscript. JMH initiated the study. BTM contributed to manuscript preparation. JMM, CMW, MS, JKT, SLM, TNP, VA performed experiments. CS performed BioPlex analysis. HIP provided MM cells. SLB helped with animal experimentation. MBM performed experiments, statistical analysis and helped with illustrations. All authors read and approved the final manuscript.

## Supplementary Material

Additional file 1: Figure S1 Asbestos causes priming and activation of NLRP3. **(A)** asbestos-induced increases in NLRP3 mRNA levels are transcriptionally regulated. LP9 were treated with Actinomycin D (Act D) for 30 min before exposing them to asbestos for 24 h. Pre-treatment with Act D resulted in inhibition of asbestos-induced NLRP3 levels. **(B)** HM3 cells exposed to asbestos (75 μm^2^/cm^2^ dish) show significantly increased *NLRP3* mRNA levels. **(C)** Increases in IL-1β and **(D)** HMGB1 in medium. (N = 2 samples/group/time point), N = 4 samples/control group (0) **(B**, **C)**. * = significantly different (p ≤ 0.05) when compared to untreated control group (0); † = significantly different (p ≤ 0.05) when compared to asbestos alone group. Click here for file

Additional file 2: Figure S2 Morphology and toxicity of pathogenic fibers. Erionite **(A)** and crocidolite asbestos **(B)** have a similar fibrous morphology (bars = 10 μm) but are vastly different in their toxicity to LP9 human mesothelial cells **(C**, **D)** (N = 3 samples/group/time point). Resistance of HMC cells to erionite toxicity may be one explanation for its increased carcinogenic potential. * = significantly different (p ≤ 0.05) when compared to untreated control group (0). **(E)** Schematic diagram illustrating mechanisms of inflammasome-induced cytokine production by asbestos and erionite in a feedback loop that is blocked by the IL-1ra, Anakinra (Ana). Click here for file
