# Supplementary figures and images for: Asbestos and erionite prime and activate the NLRP3 inflammasome that stimulates autocrine cytokine release in human mesothelial cells
Source: Part Fibre Toxicol. 2013 Aug 13;10:39. doi: 10.1186/1743-8977-10-39 (PMC3751315; doi:10.1186/1743-8977-10-39)

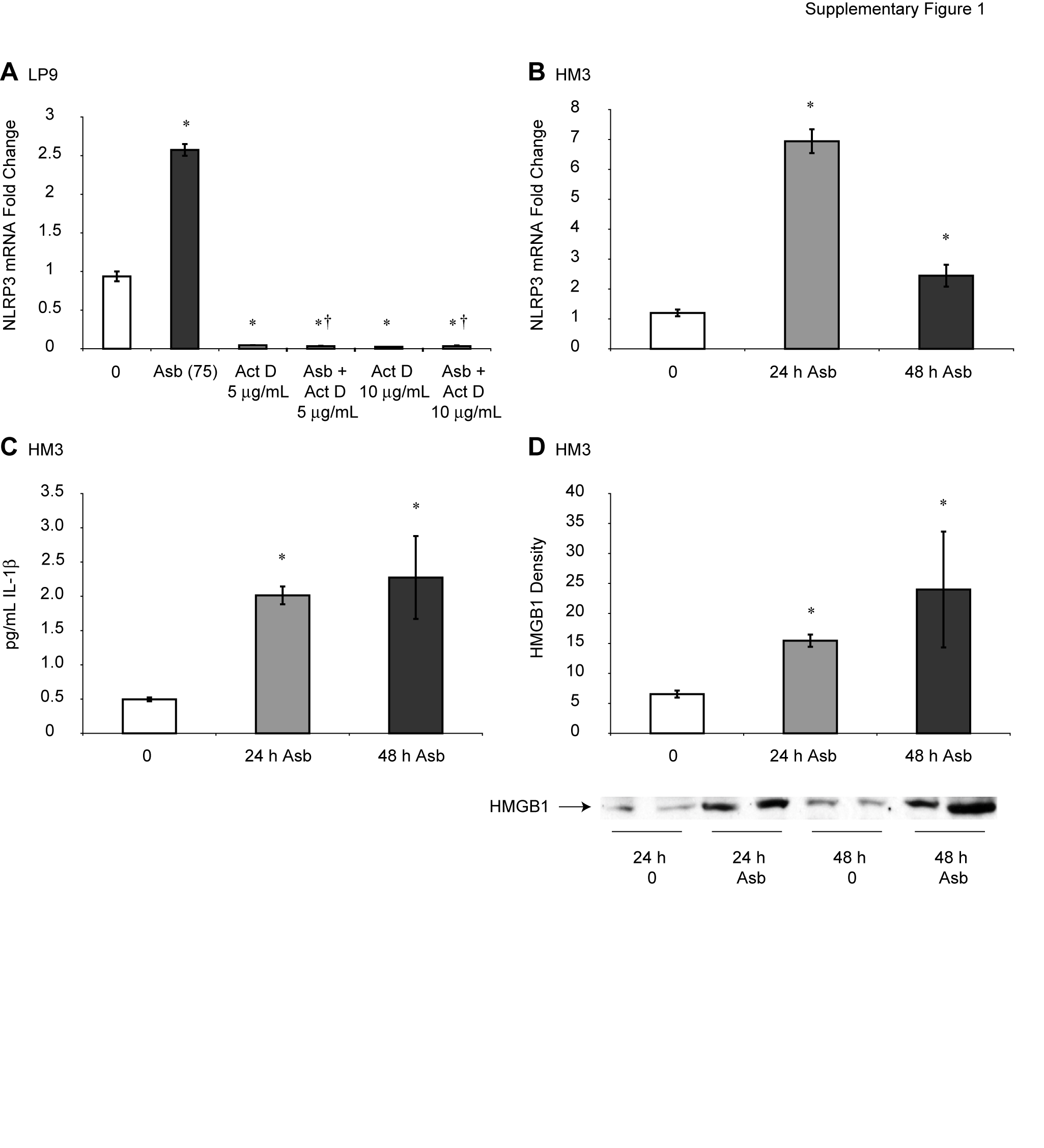

Supplement: Additional file 1: Figure S1 — Asbestos causes priming and activation of NLRP3. (A) asbestos-induced increases in NLRP3 mRNA levels are transcriptionally regulated. LP9 were treated with Actinomycin D (Act D) for 30 min before exposing them to asbestos for 24 h. Pre-treatment with Act D resulted in inhibition of asbestos-induced NLRP3 levels. (B) HM3 cells exposed to asbestos (75 μm2/cm2 dish) show significantly increased NLRP3 mRNA levels. (C) Increases in IL-1β and (D) HMGB1 in medium. (N = 2 samples/group/time point), N = 4 samples/control group (0) (B, C). * = significantly different (p ≤ 0.05) when compared to untreated control group (0); † = significantly different (p ≤ 0.05) when compared to asbestos alone group. [file 1743-8977-10-39-S1.tiff]

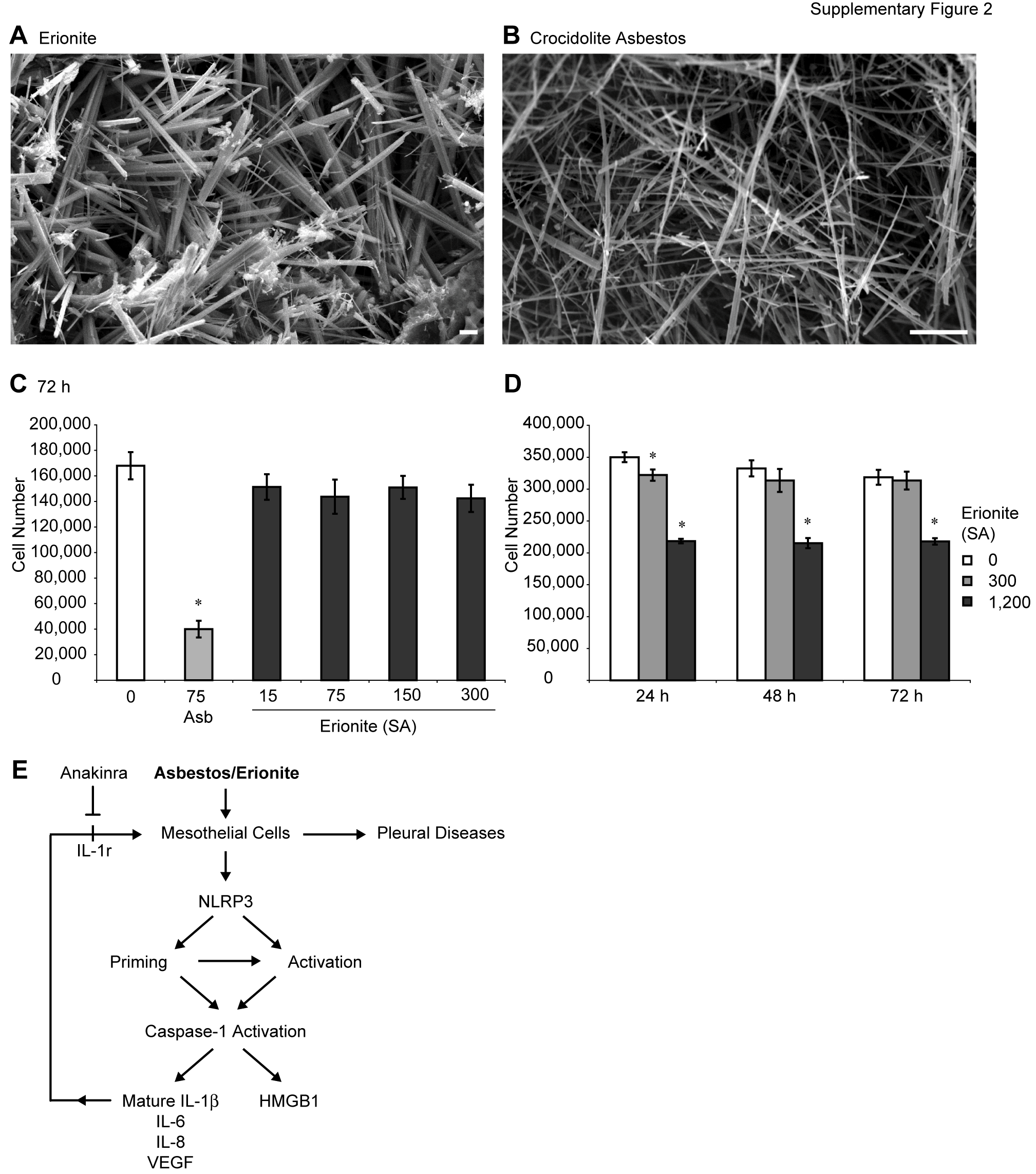

Supplement: Additional file 2: Figure S2 — Morphology and toxicity of pathogenic fibers. Erionite (A) and crocidolite asbestos (B) have a similar fibrous morphology (bars = 10 μm) but are vastly different in their toxicity to LP9 human mesothelial cells (C, D) (N = 3 samples/group/time point). Resistance of HMC cells to erionite toxicity may be one explanation for its increased carcinogenic potential. * = significantly different (p ≤ 0.05) when compared to untreated control group (0). (E) Schematic diagram illustrating mechanisms of inflammasome-induced cytokine production by asbestos and erionite in a feedback loop that is blocked by the IL-1ra, Anakinra (Ana). [file 1743-8977-10-39-S2.tiff]
